# Complete atrioventricular canal

**DOI:** 10.1186/1750-1172-1-8

**Published:** 2006-04-05

**Authors:** Raffaele Calabrò, Giuseppe Limongelli

**Affiliations:** 1 Cardiologia pediatrica, Azienda Ospedaliera Monaldi, Via Bianchi Leonardo, 80131 Napoli, Italy

## Abstract

Complete atrioventricular canal (CAVC), also referred to as complete atrioventricular septal defect, is characterised by an ostium primum atrial septal defect, a common atrioventricular valve and a variable deficiency of the ventricular septum inflow. CAVC is an uncommon congenital heart disease, accounting for about 3% of cardiac malformations. Atrioventricular canal occurs in two out of every 10,000 live births. Both sexes are equally affected and a striking association with Down syndrome was found. Depending on the morphology of the superior leaflet of the common atrioventricular valve, 3 types of CAVC have been delineated (type A, B and C, according to Rastelli's classification). CAVC results in a significant interatrial and interventricular systemic-to-pulmonary shunt, thus inducing right ventricular pressure and volume overload and pulmonary hypertension. It becomes symptomatic in infancy due to congestive heart failure and failure to thrive. Diagnosis of CAVC might be suspected from electrocardiographic and chest X-ray findings. Echocardiography confirms it and gives anatomical details. Over time, pulmonary hypertension becomes irreversible, thus precluding the surgical therapy. This is the reason why cardiac catheterisation is not mandatory in infants (less than 6 months) but is indicated in older patients if irreversible pulmonary hypertension is suspected. Medical treatment (digitalis, diuretics, vasodilators) plays a role only as a bridge toward surgery, usually performed between the 3rd and 6th month of life.

## Disease name and synonyms

Complete atrioventricular canal (CAVC); Common atrioventricular canal; Complete atrioventricular septal defect.

### European paediatric cardiac code

Reference of Complete atrioventricular canal is 06.06.09.

## Definition

CAVC is a complex cardiac malformation characterised by a variable deficiency of the atrioventricular area (crux cordis) in the developing heart. The malformation involves the atrial, ventricular and atrioventricular septa and both atrioventricular valves.

## Diagnosis criteria

**Figure 1:**
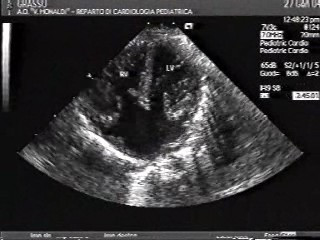
Echocardiography of Complete atrioventricular canal.

Diagnosis of CAVC might be clinically suspected in patients presenting in the first few months of life with congestive heart failure, cardiomegaly on chest X-ray and left axis deviation, bi-atrial enlargement and bi-ventricular pressure and volume overload on electrocardiogram (ECG). Echocardiography is the key tool for the diagnosis and anatomic classification of this malformation. It shows the ostium primum atrial septal defect, with the underlying common atrioventricular valve, and the defect of the ventricular septal inflow (Figure 1).

The anatomic subgroups (Rastelli's type A, B and C) can be classified on the basis of the chordal insertions and morphology of the superior bridging leaflet of the common atrioventricular valve (Table 1). Similarly, a thorough echocardiographic examination shows the degree of dysfunction of the common atrioventricular valve, as well as the presence of associated cardiac malformations. To date, cardiac catheterisation is not considered as mandatory for the diagnosis, but can be indicated in patients older than 6 months with suspected irreversible pulmonary hypertension. Cardiac catheterisation allows accurate quantification of the left-to-right shunt as well as assessment of the degree of pulmonary hypertension and the reversibility of the pulmonary artery resistances by hyperoxia and/or pharmacological tests. On left ventricular angiography, the appearance of the "gooseneck deformity" of the left ventricular outflow tract is peculiar of atrioventricular canal malformations.

**Table 1:** Anatomic classification of CAVC [4]

**Type A** the superior bridging leaflet is almost completely adherent to the left ventricle and is firmly attached on the ventricular septum by multiple chordal insertions
**Type B** the superior bridging leaflet is attached over the ventricular septum by an anomalous papillary muscle of the right ventricle
**Type C** the superior bridging leaflet is not attached to the ventricular septum (free-floating leaflet)

## Differential diagnosis

Differential diagnosis of CAVC involves mainly the unrestrictive ventricular septal defect, associated or not to mitral valve insufficiency. The clinical picture of congestive heart failure, the bi-atrial and bi-ventricular overload on ECG, and cardiomegaly and pulmonary congestion on chest X-ray are common to the ventricular septal defect. However, on ECG the left axis deviation of QRS at -30°, usually combined with a various degree of right bundle branch block, appears to be suggestive of CAVC. Eventually, the echocardiographic examination is the cornerstone for diagnosis and is of help for that of any further associated cardiac malformation.

## Epidemiology

CAVC accounts for about 3% of all cardiac malformations. Atrioventricular canal occurs in two out of every 10,000 live births. Both sexes are equally affected, with a slightly higher frequency in female (female/male ratio 1.3/1) and a striking association with Down syndrome was found [1,2].

## Pathology

The complete form of AVC shows an ostium primum atrial septal defect, a common atrioventricular valve and a variable deficiency of the interventricular septum inlet [2,3]. This anatomic arrangement gives a scooped out appearance to the ventricular inlet and a long and narrow morphology to the left ventricular outlet. The key finding for the anatomic classification in type A, B or C of this malformation is the morphology of the common atrioventricular valve [4]. It is basically built-up of five leaflets (superior, inferior, mural in the right and left ventricle and antero-superior), embryologically derived from the original endocardial cushions. The size of the antero-superior leaflet is reciprocal with the extent of bridging of the superior leaflet. In type A, the superior bridging leaflet is almost completely adherent to the left ventricle and is firmly attached on the ventricular septum by multiple chordal insertions. In type B, the superior bridging leaflet is larger and overhangs the ventricular septum more than in type A, attached over it by an anomalous papillary muscle of the right ventricle. In type C, the superior bridging leaflet is larger and is not attached to the ventricular septum (free-floating leaflet), thus provoking an unrestricted interventricular communication. Type A CAVC is most frequently associated with left-sided obstructions. Type B is the least common form of atrioventricular canal. Type C is often associated with other complex cardiac malformations such as tetralogy of Fallot. Other cardiac malformations are the left ventricular inflow and outflow obstructions, mainly due to anomaly of the left component of the common atrioventricular valve, and to ventricular imbalance, with right ventricular dominance. These additional left-sided anomalies are more frequent in children without Down syndrome [5-7].

A "partial variant" of AVC exists (also known as ostium primum atrial septal defect). Nevertheless, some authors observed how "complete" and "partial" are inappropriate adjectives to describe these variants [8-11]. Indeed, despite the septal deficiency, the essence of the atrioventricular canal malformations (or atrioventricular septal defects) is the common atrioventricular junction [8-11]. Within this common junction, there may be a common atrioventricular valvar orifice (so called "complete" defects), or separate right and left valvar orifices for the right and left ventricles (so called "partial" defects).

The space between the left ventricular components of the superior and inferior bridging leaflets is traditionally called "cleft". Morphological studies suggested that this gap functions as a commissure, even though it is not supported by a papillary muscle [12]. In Rastelli's type A malformation, the space in the common anterior leaflet is also called "cleft". Again, it potentially functions as a commissure, being supported by the medial papillary muscle of the right ventricle [12].

## Clinical description

Symptoms occur in infancy as a result of high pulmonary blood flow associated with pulmonary hypertension, and often complicated by insufficiency of the common atrioventricular valve. Failure to thrive, as well as congestive heart failure and frequent pulmonary infections, are invariably seen. Thus, patients with CAVC often have feeding problems and are virtually symptomatic in the first few months of life. Signs of congestive heart failure consist in feeding difficulties, excessive sweating, tachycardia, tachypnea, subcostal and intercostal retractions, mild wheezing, hepatic enlargement and poor peripheral blood perfusion [13]. If a significant regurgitation of the common atrioventricular valve is present, a systolic cardiac murmur and gallop rhythm are frequently heard. Over time, irreversible pulmonary hypertension develops, improving the signs of congestive heart failure but worsening tolerance to effort. When pulmonary artery resistances becomes higher than systemic artery resistances, the intracardiac shunt reverses and cyanosis develops, further decreasing the exercise capacity.

## Natural history

Half of children with untreated CAVC die in the first year of life [1,13,14]. The main cause of death in infancy is either heart failure or pneumonia. In surviving patients with unrepaired complete atrioventricular canal, irreversible pulmonary vascular disease becomes increasingly common, and affects virtually all patients older than 2 years of age [15]. Long-term prognosis in patients with irreversible pulmonary hypertension is poor.

## Treatment

### Medical treatment

Medical therapy aims to improve the signs and symptoms of congestive heart failure. Thus, it should be just considered as a bridge toward surgery. Pharmacological therapy is based on digitalis, diuretics and vasodilators. Oral therapy with digoxin starts with a loading dose of 20–40 **μ**g/kg (depending on the patient's age, from premature to child) in 3 doses over 24 h, continuing with a maintenance dose of 8–10 **μ**g/kg/day in 2 doses. Diuretic therapy is mainly based on furosemide, at the dose of 1–6 mg/kg/day, and spironolactone, at the dose of 2–3.5 mg/kg/day. Vasodilator therapy consists chiefly in the angiotensin converting enzyme inhibitors, captopril (0.5–3 mg/kg/day, tid) or enalapril (0.1–0.4 mg/kg/day, bid). Increasing interest is raising regarding the utilisation and potential benefits of beta-blockers (mainly, propanolol, metoprolol and carvedilol) in infants and children heart failure due to congenital heart defects with left to right shunt, although long-term results are needed. Finally, the new generation of pulmonary vasodilators dramatically improved the post-operative course and the overall prognosis of the patients [16].

### Surgical treatment

Surgical treatment is preferably scheduled before 6–12 months of life. Generally, the great majority of surgeons perform the repair between the 3rd (to reduce the incidence of pulmonary hypertension crisis) and the 6th month of life. Surgical palliation with pulmonary artery banding is now seldom indicated in high-risk infants (very low weight and/or in critical conditions). It reduces the pulmonary artery flow and pressure, so controlling the congestive heart failure, promoting the patient's growth and preventing the development of pulmonary vascular disease, but is contra-indicated in patients with severe atrioventricular valve regurgitation. However, more frequently complete intracardiac repair is indicated. It consists in closure of the intracardiac communications with a single or separate atrial and ventricular patches, in construction of two separate and competent atrioventricular valves using the available tissue from the common atrioventricular valve leaflet, and in repair of associated cardiac anomalies [13,17,18]. An alternative technique, using a direct suture closure of the ventricular component, accompanied by pericardial patch closure of the atrial component, was first suggested by Wilcox *et al*. [19]. Depending on the specific anatomic findings (*i.e*., in absence of severe "scooping" of the ventricular septum), the lesion can be adequately repaired in most instances by sewing down the bridging leaflets to the crest of the ventricular septum.

Risk factors for surgical repair include the patient's age, the severity of pre-operative common valve incompetence, the presence of associated cardiac malformations and the degree of the functional class [20][21][22][23]. The prognosis is directly related to the repair of the left A-V valve [20]. To date, the overall mortality for primary repair of CAVC is below 5–10%. Long-term survival is good and in 80%–95% of cases there is no need for reoperation [18,21]. Of note, the closure of the cleft results in longer times before a reoperation is necessary [24].

## Aetiology

Formation of atrioventricular canal results from complex interactions of components of the extracellular matrix. Septation of the atrioventricular junction is brought about by downgrowth of the primary atrial septum, fusion of the endocardial cushions and forward expansion of the vestibular spine between atrial septum and cushions [3]. Thus, atrioventricular canal can result from arrest or interruption of the normal endocardial cushion development [25,26]. Experimental studies showed that environmental teratogens [27] or endogenous metabolic abnormalities [28] might result in abnormal development of the atrioventricular area, which may be due to altered apoptosis of these forming cells [29]. Trancription factors (TBX2, Foxp1 among the others) and signal pathways (ErbB receptor activation) involved during embryogenesis in the heart development process have been strongly suggested to have a role in atrioventricular septation [30-32].

Epidemiological studies [2,33] showed that complete atrioventricular canal tends to be associated with chromosomal abnormalities, mainly Down syndrome [10], del [8p] syndrome [34], trisomy 9, trisomy 18 [6,35]. Furthermore, CAVC with Down syndrome has been less frequently associated with left cardiac anomalies than the isolated form [5,7]. In this latter subset of patients, the analysis of potential risk factors revealed an association with maternal diabetes and antitussive drugs [36]. However, in patients with Down syndrome and complete AVC, no strong association other than maternal age has been found. In the asplenia syndrome, the CAVC is virtually always present, while it occurs in about 25% of patients with polisplenia [37]. An association between nonchromosomal defects and atrioventricular malformations have also been reported [5,6].

## Genetic counselling and antenatal diagnosis

In the presence of a single affected family member, the risk of siblings of inheriting the defect is about 2%, with a higher percentage for the offspring of an affected parent. Concordance for atrioventricular malformations among siblings is higher than for other types of congenital heart defects [38].

Due to the strict association with Down syndrome and other chromosomal anomalies, genetic antenatal counselling after the foetal echocardiographic diagnosis of CAVC is mandatory. At present, prenatal diagnosis of CAVC has been associated with a 58% risk of aneuploidy, mainly trisomy 21 [39]. Again, due to the strong association between chromosomal abnormalities and CAVC, when this malformation seems isolated at antenatal echocardiography, the risk of trisomy 21 is significantly higher than when other associated cardiac lesions are diagnosed.
